# Anorexia Nervosa and Autism Spectrum Disorder: what links them?

**DOI:** 10.1192/j.eurpsy.2023.1110

**Published:** 2023-07-19

**Authors:** C. M. Santos, A. Quintão, D. Jeremias, M. Fraga

**Affiliations:** 1 Psychiatry and Mental Health, Centro Hospitalar de Lisboa Ocidental, Lisbon; 2 Psychiatry and Mental Health, Hospital de Cascais, Cascais, Portugal

## Abstract

**Introduction:**

According to the literature, about 35% of patients with Anorexia Nervosa (AN) also have a diagnosis of Autism Spectrum Disorder (ASD), and this comorbidity occurs more frequently in males.

**Objectives:**

With this work, the authors intend to address the characteristics present in this comorbidity and what is the impact of this comorbidity in the diagnosis, approach and prognosis of AN.

**Methods:**

Non-systematic research of the literature through the PubMed database with the terms “autism spectrum disorder” and “anorexia nervosa”. Only surveys conducted in the last 10 years were considered for inclusion.

**Results:**

Although AN and ASD may seem to be quite distinct conditions, the studies found suggest the existence of four characteristics that overlap the two diagnoses: deficits in theory of mind, inability to switch between courses of action fluently, inability to see the whole pictures to the detriment of detail and alexithymia. Studies also point to greater resistance to treatment in AN when an ASD is present in comorbidity.

**Conclusions:**

Scientific evidence suggests that autistic characteristics in people with AN are not a consequence of being underweight, but rather stable characteristics present before and after the onset of AN. The studies thus conclude that comorbidity between the two disorders exists and is frequent enough to warrant greater attention to the diagnosis of ASD in people with AN. However, there are still no specific guidelines for the treatment of AN in people with ASD, which leads to a worse response to treatment, evolution and prognosis of AN in people with ASD.

**Disclosure of Interest:**

None Declared

